# Hepatic artery pseudoaneurysm, bronchobiliary fistula in a patient with liver trauma

**DOI:** 10.1186/s12893-018-0437-9

**Published:** 2018-11-12

**Authors:** Prabhat Jha, Bijendra Dhoj Joshi, Binit Kumar Jha

**Affiliations:** 1 Alka Hospital Private Limited, Pulchowk, Lalitpur Nepal; 2 Department of Surgery, Kathmandu Model Hospital, Kathmandu, Nepal; 3 Department of Surgery, National Academy of Medical Sciences, Kathmandu, Nepal

**Keywords:** Liver trauma, Bronchobiliary fistula, Pseudoaneurysm

## Abstract

**Background:**

Bronchobiliary fistula and hepatic artery pseudoaneurysm are rare complications of hepatic trauma. There are isolated case reports for both pseudoaneurysm and bronchobiliary fistula following hepatic trauma but there aren’t reports of both conditions developing in a single patient.

**Case presentation:**

This case describes an 18 year old hindu male who developed right hepatic artery pseudoaneurysm and bronchobiliary fistula following blunt abdominal trauma. Patient was managed with exploratory laparotomy followed by coil embolization and Endoscopic retrograde cholangiopancreatography stenting respectively.

**Conclusion:**

Rare complications of liver trauma include pseudoaneurysm and bronchobiliary fistula. These complications can rarely co- exist in a single patient.

## Background

Bronchobiliary fistula is a rare disorder, first reported by Peacock in 1850 [1]. It consists of an abnormal communication between the biliary tract and bronchial tree. Bronchobiliary fistulas are usually caused by hepatic or subphrenic abscesses, resulting from different conditions and rarely as a complication of liver injury. It is a rare cause of chronic cough leading to biliptysis [1]. It is usually diagnosed by clinical history and imaging (Computed tomography/Magnetic Resonance Cholangiography) [2–5]. Treatment is endoscopic retrograde cholangiopancreatography and stenting or surgery [6, 7].

Hepatic artery pseudoaneurysm following liver trauma is also a rare complication occurring in 4% of patients and is a cause for delayed hemorrhage and hemobilia [8]. Symptoms of an hepatic artery pseudoaneurysm varies from clinically silent to signs of rupture with intra-peritoneal haemorrhage or rupture into the gastrointestinal tract, venous, portal or biliary system. The management for psuedoaneurysm has shifted from more aggressive open approach to endovascular techniques [9].

There are isolated case reports of hepatic artery pseudoaneurysm and bronchobiliary fistula following trauma but there aren’t any case reports of both conditions occurring in a single patient.

The information presented in this case report have been obtained from the patient’s hospital records. Written informed consent was taken from the patient for publication of this case report. Regarding availability of data and materials all relevant images including that of contrast enhanced computed tomography of chest and abdomen, images of angiography and coil embolization, image of endoscopic retrograde cholangiopancreatography have been uploaded along with the main manuscript. For the sake of confidentiality, pertinent identifiers have been omitted from the images.

## Case presentation

**Fig. 1:**
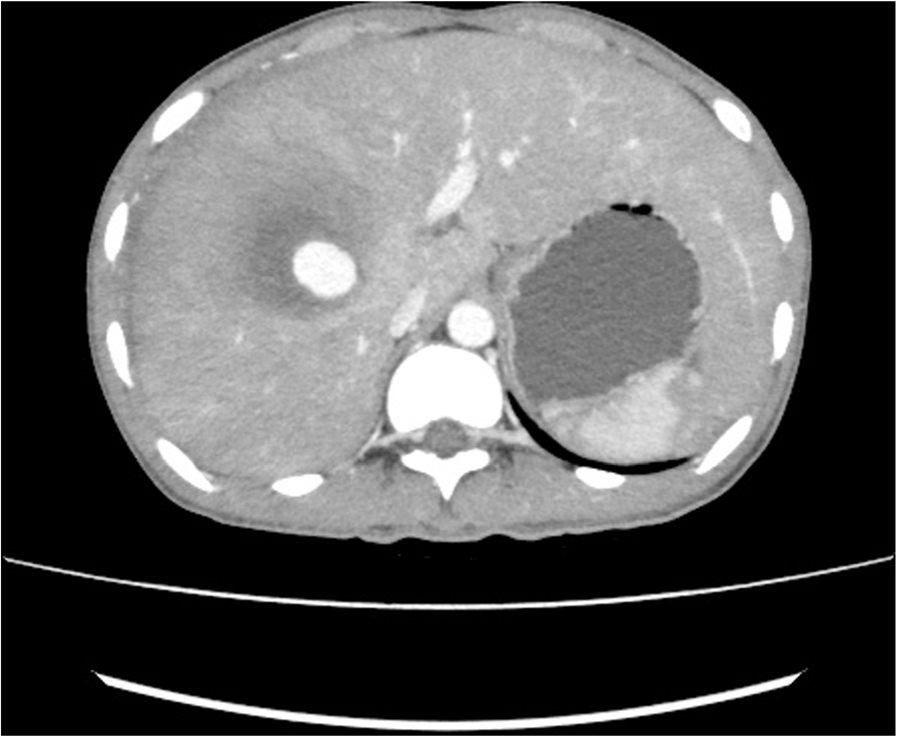
CECT showing hepatic artery pseudoaneurysm

**Fig. 2:**
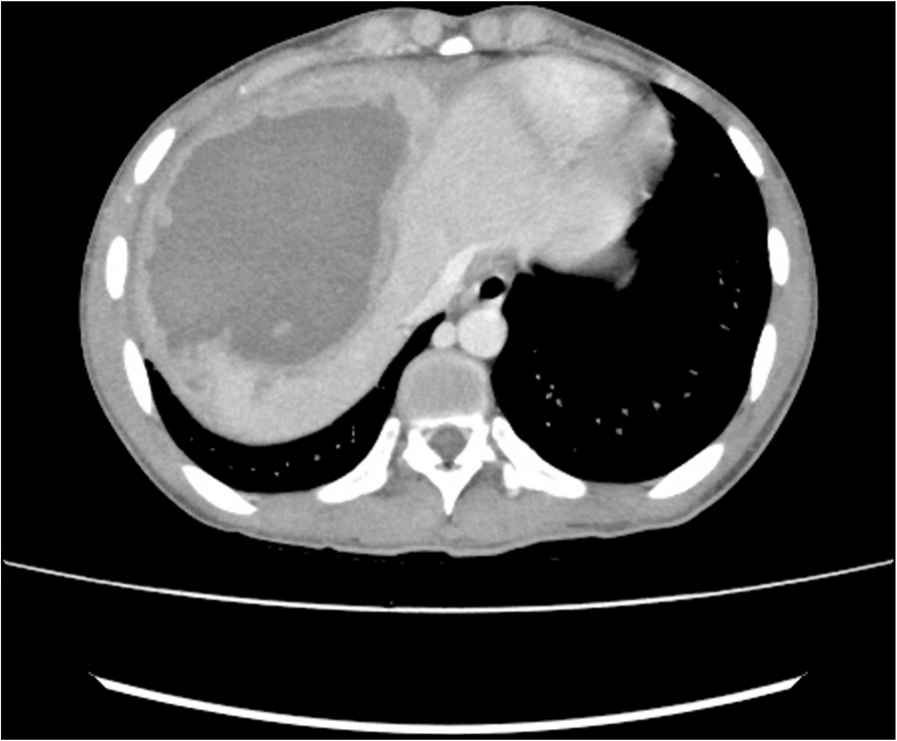
CECT showing liver hematoma

An 18 year old male fell from roof of a moving truck. The truck was moving at low speed and he fell on muddy floor from a height of approximately 12 feet and sustained impact over abdomen. He was admitted in Nepalgunj medical college where contrast enhanced computed tomography of abdomen showed grade III liver injury. He was managed conservatively with bed rest, 12 units of blood transfusion then discharged. He presented 15 days later to Alka Hospital emergency department with complaints of pain abdomen, excessive tiredness and vomiting of blood. His vitals in the emergency were pulse 100/min, blood pressure 90/60 mmHg, respiratory rate 20/min and temperature 99 degree F. Contrast enhanced computed tomography of abdomen was done which showed pseudoaneurysm of right hepatic artery with ruptured hematoma in segments VI, VII and VIII of liver (Figs. 1 and 2). Clot was present in the pelvis and lesser sac. Haemoglobin was 8 g/dL.

Patient was immediately transferred to intensive care unit and started on intravenous fluid, antibiotics. Blood transfusion was started but the patient became hemodynamically unstable. Patient was transferred to operation theatre and exploratory laparotomy was done. Intraoperative finding was approximately three litres of clot and blood in lesser sac and pelvis with approximately 5 cm laceration in segments VII and VIII of liver. Bleeding from the liver surface was controlled with gel foam packing, surgicell application. Drains were kept in Morrison’s pouch and pelvis. Five units of packed cell and two units of fresh frozen plasma was transfused intraoperatively. The pseudoaneurysm couldn’t be clipped intraoperatively due to the difficult location and hemodynamic instability of the patient. Postoperatively patient was transferred to intensive care unit. Patient was transfused with two units of blood postoperatively. Patient was transferred to the general ward on third postoperative day. Patient was discharged on eighth postoperative day.

**Fig. 3:**
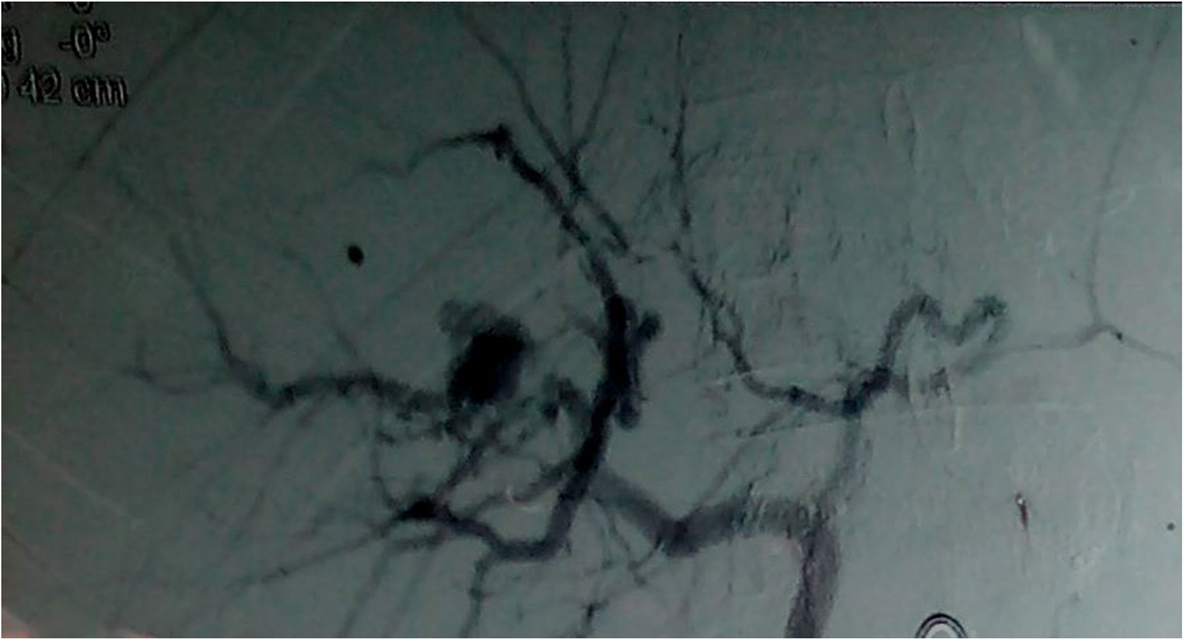
Hepatic artery pseudoaneurysm in angiogram

**Fig. 4:**
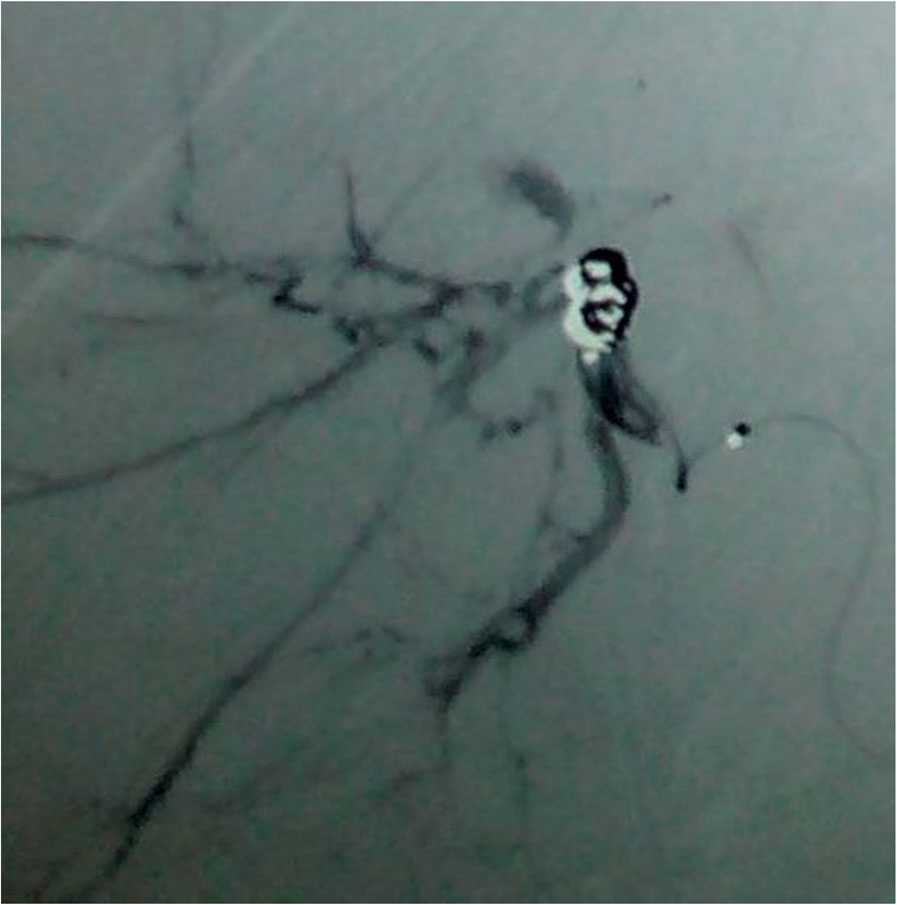
Post coiling of pseudoaneurysm

On the 15th post operative day patient presented with hematemesis two episodes. On presentation to the out patient department, he was hemodynamically stable. CECT abdomen revealed hematoma in right lobe of liver with right hepatic artery pseudoaneurysm. Upper gastrointestinal endoscopy was done which confirmed hemobilia. Coil embolization of the pseudoaneurysm was planned and he was transferred to the interventional radiology unit in Tribhuvan University Teaching Hospital. Under aseptic precaution using femoral artery access coil embolization of hepatic artery pseudoaneurysm was done. Three coils were used for embolization (Figs. 3 and 4).

**Fig. 5:**
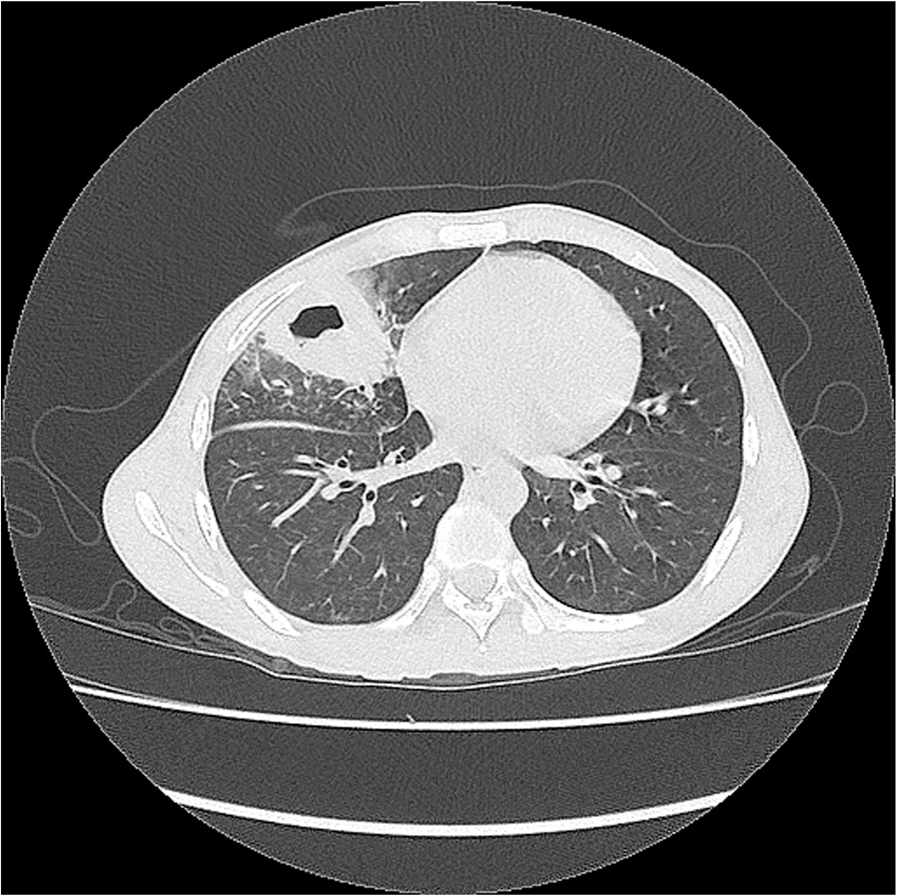
CECT showing right lung basal consolidation at fistula site

On the third post embolization day, patient developed high grade fever and persistent cough. Chest examination revealed basal crepitations on right side. Chest X ray and contrast enhanced computed tomography chest showed right lower lobe consolidation (Fig. 5). Patient was started on intravenous antibiotics but the cough didn’t subside. Sputum content was sanguinous. On the seventh post embolisation day patient coughed up bile and a diagnosis of post traumatic bronchobiliary fistula was made. Bilioptysis increased with supine position. Endoscopic Retrograde Cholangiopancreatography was performed which showed an area of bile leak at right hepatic lobe close to the diaphragm at right anterior duct. Contrast was seen spilling into the right bronchial tree confirming fistulation. No obvious tract was noted. Modest sphincterotomy was performed and 10 Fr double pigtail stent was deployed. Post stenting the cough and bilioptysis resolved and the patient was discharged on the third day post endoscopic retrograde cholangiopancreatography. The stent was removed at 2 months. Follow up ultrasound scan at 2 months showed complete resolution of the hematoma.

## Discussion and conclusion

Bronchobiliary fistula and hepatic artery pseudoaneurysm following liver trauma are both rare complications of liver trauma. There are isolated reports for both conditions but this case is interesting because of the fact that both conditions were present in the same patient.

Bronchobiliary fistula is a rare disorder usually caused by liver abscess [10], trauma [11], hepatic hydatids [7], hepatic tumors, following radiofrequency thermal ablation of hepatic tumors [12], post liver resection [13], chronic pancreatitis [14] and rarely as a late complication of transcatheter arterial embolisation. In this case the cause of fistula was trauma. The pathogenesis involves biliary obstruction and presence of severe inflammation close to the primary lesion [15].

The usual presentation of patients is chronic intractable cough, biliptysis, fever, and pain. The cough is postural and gets aggravated in supine position [4, 5, 15]. The condition can initially be misdiagnosed as pneumonia as in this case. Bilioptysis usually confirms the diagnosis as in this case.

Magnetic resonance cholangiography may reveal a communication between the biliary tree and the bronchial tree [2]. Hepatobiliary scintigraphy can also be used as a noninvasive means for the precise diagnosis of a bronchobiliary fistula [3]. Endoscopic retrograde cholangiopancreatography showed a fistulous communication between the biliary and bronchial tree in our case.

The first line of treatment is endoscopic. Endoscopic biliary sphincterotomy and repeated insertion of large size biliary plastic stents have been used successfully for treatment of the fistula [6]. In this case as well endoscopic retrograde cholangiopancreatography with insertion of biliary stent was done and it was successful. Surgical intervention is reserved for patients in whom endoscopic treatment has failed. Surgery involves resection of involved pulmonary tissue and interposition of viable tissue between lung and the fistulous tract [7].

Hepatic artery pseudoaneurysm following liver trauma is quite rare occurring in 4% of patients with risks of rupture and hemobilia [8]. It is a false aneurysm that develops from a leakage of an injured artery into the surrounding tissues forming a cavity outside the artery. It can be distinguished from a haematoma as it continues to communicate with the artery resulting in a high-pressure cavity with the risk of rupture. The most common cause is trauma but it can also occur after hepato-biliary surgery, pancreatitis, gallstone, percutaneous procedures such as liver biopsy [16, 17]. Pseudoaneurysm are thought to be the result of direct trauma. Symptoms can vary from clinically silent to intraperitoneal rupture. Management usually involves prophylactic angioembolization of the pseudoaneurysm if detected on follow up CT scan [16].

In conclusion bronchobiliary fistula and hepatic artery pseudoaneurysm can rarely occur together in patients with traumatic liver injury. Bronchobiliary fistula can be misdiagnosed and treated as pneumonia. Both conditions can be treated with minimally invasive modalities.
